# Supplementary material for: Glucosamine Use Is Associated with a Higher Risk of Cardiovascular Diseases in Patients with Osteoarthritis: Results from a Large Study in 685,778 Subjects
Source: Nutrients. 2022 Sep 7;14(18):3694. doi: 10.3390/nu14183694 (PMC9506459; doi:10.3390/nu14183694)
Supplement: Supplementary file 1 [file nutrients-14-03694-s001.zip › nutrients-1884392-supplementary.pdf]

Supplementary Materials

Table S1. The distribution of period time from diagnosis of osteoarthritis to starting taking glucosamine

Table S2. The items and weights in Charlson Comorbidity Index (CCI)

Table S3. Incidence of cardiovascular disease (CVD) events

Table S4. Association of glucosamine use with cardiovascular disease (CVD) in subgroups

Table S5. Characteristics of the study population after 1:2 propensity score matching

Table S6. Incidence of CVD events after 1:2 propensity score matching

Table S7. Association of glucosamine use with CVD adjusting for adherence of antidiabetic drugs

Table S8. Association of glucosamine use with CVD excluding patients with <2 years follow-up period

Table S9. Association of glucosamine use with CVD including all patients newly diagnosed with osteoarthritis.

Table S10. Association of glucosamine use with CVD from the diagnosis of osteoarthritis to the outcomes.

Table S1. The distribution of period time from diagnosis of osteoarthritis to starting taking glucosamine.

T	Glucosamine users, *n* (%)			
	**Non-adherent**	**Partially adherent**	**Adherent**	**Overall**
**0 ***	93,050 (48.21)	16,604 (42.17)	3,502 (43.65)	113,156 (47.07)
**<1 month**	18,820 (9.75)	4,343 (11.03)	1,062 (13.24)	24,225 (10.08)
**1-3months**	21,232 (11.00)	5,199 (13.20)	1,077 (13.42)	27,508 (11.44)
**3-6months**	23,037 (11.93)	5,356 (13.60)	1,065 (13.27)	29,458 (12.25)
**6-12months**	36,885 (19.11)	7,870 (19.99)	1,317 (16.42)	46,072 (19.16)

T indicated the period time from diagnosis of osteoarthritis to starting taking glucosamine. The values were all 0 in glucosamine non-users. The HRs were adjusted for age, sex, hypertension, medication, Charlson Comorbidity Index (CCI), and health care utilization index (HCUI). * "0" indicated starting glucosamine immediately after the diagnosis of osteoarthritis.

Table S2. The items and weights in Charlson Comorbidity Index (CCI).

The items in CCI	Scores (Weights)
Myocardial infarction	1
Congestive heart failure	1
Peripheral vascular disease	1
Cerebrovascular disease	1
Dementia	1
Chronic pulmonary disease	1
Connective tissue disease	1
Ulcer disease	1
Mild liver disease	1
Diabetes	1
Hemiplegia	2
Moderate or severe renal disease	2
Diabetes with end organ damage	2
Any tumor without metastasis	2
Leukemia	2
Lymphoma	2
Moderate or severe liver disease	3
Metastatic solid tumor	6
AIDS	6

Table S3. Incidence of cardiovascular disease (CVD) events.

	Overall CVD		CHD		Stroke	
	**No. of events**	**Incidence per 1,000 person-years (95% CI)**	**No. of events**	**Incidence per 1,000 person-years (95% CI)**	**No. of events**	**Incidence per 1,000 person-years (95% CI)**
Non-glucosamine	40,858	15.08 (14.93, 15.23)	16,686	6.01 (5.91, 6.10)	11,742	4.21 (4.13, 4.28)
Glucosamine	23,742	16.63 (16.42, 16.84)	9,844	6.72 (6.59, 6.85)	6,090	4.13 (4.03, 4.23)
Non-adherent	17,751	15.47 (15.24, 15.70)	7,399	6.30 (6.16, 6.44)	4,554	3.85 (3.74, 3.97)
Partially adherent	4,657	19.99 (19.41, 20.56)	1,874	7.78 (7.43, 8.14)	1,183	4.88 (4.60, 5.15)
Adherent	1,334	28.18 (26.67, 29.69)	571	11.47 (10.53, 12.41)	353	7.00 (6.27, 7.73)

CVD, cardiovascular disease; CHD, coronary heart disease; CI, confidence interval.

Table S4. Association of glucosamine use with cardiovascular disease (CVD) in subgroups.

Subgroup	HR (95% CI) for CVD			*P* for trend
	Non-adherent	Partially adherent	Adherent	
All	1.03 (1.02, 1.05)	1.26 (1.22, 1.30)	1.68 (1.59, 1.78)	<0.001
Age				
<60 years	1.00 (0.97, 1.03)	1.12 (1.06, 1.19)	1.62 (1.46, 1.80)	<0.001
≥60 years	1.02 (1.00, 1.04)	1.23 (1.19, 1.28)	1.59 (1.49, 1.70)	<0.001
Sex				
Female	1.03 (1.01, 1.06)	1.25 (1.19, 1.30)	1.76 (1.64, 1.89)	<0.001
Male	1.06 (1.03, 1.09)	1.31 (1.26, 1.38)	1.62 (1.49, 1.76)	<0.001

NSAIDs, non-steroidal anti-inflammatory drug; CCI, Charlson Comorbidity Index; HCUI, health care utilization index; HCUI, health care utilization index; HR, hazard ratios; CI, confidence interval. The HRs were adjusted for age, sex, hypertension, medication, Charlson Comorbidity Index (CCI), and health care utilization index (HCUI).

Table S5. Characteristics of the study population after 1:2 propensity score matching.

Characteristics	Glucosamine non-users (*n*= 16,043)	Adherent glucosamine users (*n*= 8,022)	*P* value
Age, years, mean (SD)	62.02±14.04	61.89±10.81	0.48
Female, *n* (%)	10586 (65.99)	5316 (66.27)	0.66
Hypertension, *n* (%)	3991 (24.88)	2045 (25.49)	0.30
Lipid treatment, *n* (%)	7844 (48.89)	3930 (48.99)	
Antiplatelet treatment, *n* (%)	6132 (38.22)	3100 (38.64)	0.53
NSAIDs therapy, *n* (%)	5113 (31.87)	2562 (31.94)	0.92
CCI, mean (SD)	0.80±1.03	0.80±0.87	0.99
HCUI, median (IQR)	17 (6, 36)	14 (3, 36)	0.81

NSAIDs, non-steroidal anti-inflammatory drug; CCI, Charlson Comorbidity Index; HCUI, health care utilization index; IQR, inter quartile range. The propensity scores for each participant between groups were calculated using a logistic regression model including the index year and all covariates.

Table S6. Incidence of CVD events after 1:2 propensity score matching.

	Glucosamine non-users (*n*= 16,043)		Adherent glucosamine users (*n*= 8,022)		HR (95%CI)	*P* value
	Number of events	Incidence (95% CI)	Number of events	Incidence (95% CI)		
Overall CVD	1,978	20.48 (19.58, 21.38)	1,333	28.16 (26.65, 29.67)	1.38 (1.29, 1.48)	<0.001
CHD	790	7.89 (7.34, 8.45)	570	11.45 (10.51, 12.39)	1.45 (1.30, 1.62)	<0.001
Stroke	596	5.93 (5.45, 6.40)	353	7.00 (6.27, 7.73)	1.18 (1.03, 1.34)	0.016

CVD, cardiovascular disease; CHD, coronary heart disease; HR, hazard ratios; CI, confidence interval.

Table S7. Association of glucosamine use with CVD adjusting for adherence of antidiabetic drugs.

Outcomes	HR (95% CI)	*P* value	HR (95% CI)		
			Non-adherent	Partially adherent	Adherent
CVD	1.10 (1.08, 1.11)	<0.001	1.04 (1.02, 1.06)	1.25 (1.21, 1.29)	1.63 (1.54, 1.72)
CHD	1.12 (1.09, 1.15)	<0.001	1.07 (1.04, 1.10)	1.22 (1.17, 1.28)	1.64 (1.51, 1.78)
Stroke	1.03 (1.00, 1.06)	0.091	0.98 (0.94, 1.01)	1.15 (1.08, 1.22)	1.48 (1.33, 1.65)

CVD, cardiovascular disease; CHD, coronary heart disease; CI, confidence interval. The HRs were adjusted for age, sex, hypertension, medication, Charlson Comorbidity Index (CCI), health care utilization index (HCUI), and adherence of antidiabetic drugs.

Table S8. Association of glucosamine use with CVD excluding patients with <2 years follow-up period.

Outcomes	HR (95% CI)	*P* value	HR (95% CI)		
			Non-adherent	Partially adherent	Adherent
CVD	1.10 (1.08, 1.11)	<0.001	1.07 (1.05, 1.09)	1.22 (1.18, 1.26)	1.58 (1.48, 1.68)
CHD	1.12 (1.09, 1.15)	<0.001	1.1 0(1.07, 1.13)	1.19 (1.13, 1.25)	1.62 (1.48, 1.79)
Stroke	1.03 (0.99, 1.06)	0.11	1.01 (0.98, 1.05)	1.12 (1.05, 1.2)	1.37 (1.21, 1.55)

CVD, cardiovascular disease; CHD, coronary heart disease; CI, confidence interval. The HRs were adjusted for age, sex, hypertension, medication, Charlson Comorbidity Index (CCI), and health care utilization index (HCUI).

Table S9. Association of glucosamine use with CVD including all patients newly diagnosed with osteoarthritis.

Outcomes	HR (95% CI)	*P* value	HR (95% CI)		
			Non-adherent	Partially adherent	Adherent
CVD	1.15 (1.13, 1.17)	<0.001	1.09 (1.07, 1.11)	1.33 (1.30, 1.37)	1.75 (1.66, 1.84)
CHD	1.17 (1.14, 1.20)	<0.001	1.12 (1.09, 1.14)	1.32 (1.26, 1.37)	1.72 (1.60, 1.86)
Stroke	1.08 (1.05, 1.11)	<0.001	1.03 (1.00, 1.06)	1.22 (1.16, 1.28)	1.61 (1.46, 1.77)

CVD, cardiovascular disease; CHD, coronary heart disease; CI, confidence interval. The HRs were adjusted for age, sex, hypertension, medication, Charlson Comorbidity Index (CCI), and health care utilization index (HCUI). All patients newly diagnosed with osteoarthritis were included whether or not they took glucosamine within one year.

Table S10. Association of glucosamine use with CVD from the diagnosis of osteoarthritis to the outcomes.

Outcomes	HR (95% CI)	*P* value	HR (95% CI)		
			Non-adherent	Partially adherent	Adherent
CVD	1.11 (1.09, 1.13)	<0.001	1.05 (1.03, 1.07)	1.27 (1.23, 1.31)	1.70 (1.61, 1.81)
CHD	1.13 (1.10, 1.16)	<0.001	1.08 (1.05, 1.12)	1.24 (1.18, 1.31)	1.72 (1.57, 1.88)
Stroke	1.04 (1.00, 1.07)	0.044	0.98 (0.95, 1.02)	1.17 (1.10, 1.25)	1.54 (1.37, 1.73)

CVD, cardiovascular disease; CHD, coronary heart disease; CI, confidence interval. The HRs were adjusted for age, sex, hypertension, medication, Charlson Comorbidity Index (CCI), and health care utilization index (HCUI). The follow-up time was re-defined separately as the period from the diagnosis of osteoarthritis to the outcomes.
